# Fast optimization of statistical potentials for structurally constrained phylogenetic models

**DOI:** 10.1186/1471-2148-9-227

**Published:** 2009-09-09

**Authors:** Cécile Bonnard, Claudia L Kleinman, Nicolas Rodrigue, Nicolas Lartillot

**Affiliations:** 1 Département d'Informatique, LIRMM, 161 rue Ada, 34392 Montpellier Cedex 5, France; 2 Département de Biochimie, Université de Montréal, Montréal, Québec, Canada; 3 Department of Biology, University of Ottawa, Ottawa, Ontario, Canada

## Abstract

**Background:**

Statistical approaches for *protein design *are relevant in the field of molecular evolutionary studies. In recent years, new, so-called structurally constrained (*SC*) models of protein-coding sequence evolution have been proposed, which use statistical potentials to assess sequence-structure compatibility. In a previous work, we defined a statistical framework for optimizing knowledge-based potentials especially suited to SC models. Our method used the maximum likelihood principle and provided what we call the *joint *potentials. However, the method required numerical estimations by the use of computationally heavy *Markov Chain Monte Carlo *sampling algorithms.

**Results:**

Here, we develop an alternative optimization procedure, based on a *leave-one-out *argument coupled to fast gradient descent algorithms. We assess that the leave-one-out potential yields very similar results to the joint approach developed previously, both in terms of the resulting potential parameters, and by Bayes factor evaluation in a phylogenetic context. On the other hand, the leave-one-out approach results in a considerable computational benefit (up to a 1,000 fold decrease in computational time for the optimization procedure).

**Conclusion:**

Due to its computational speed, the optimization method we propose offers an attractive alternative for the design and empirical evaluation of alternative forms of potentials, using large data sets and high-dimensional parameterizations.

## Background

Recent advances in computer science and in the acquisition of new genetic sequences from a variety of organisms have opened up a wide spectrum of new possibilities in molecular evolutionary modeling. In particular, codon substitution models explicitly formulated in terms of a balance between mutation and selection constitute an attractive strategy [1-4]. By deriving the substitution process from basic principles of population genetics, their aim is to bridge the gap between population genetics and phylogenetics, and thus to offer a better understanding of the driving forces of the long term evolutionary process. More specifically, these mutation-selection models propose that the substitution rate from a sequence *s *to another *s' *(*R*_*ss'*_) depends on the rate of mutation from *s *to *s' *(), and on the probability for this mutation to be fixed in the population (*p*_*fix*_(*ss'*)):

(1)

The mutation matrix  depends only on the underlying mutation model, and is generally assumed to be fixed along the lineages and uniform along the sequence. The fixation probability *p*_*fix*_(*ss'*) depends on the particular model chosen.

Among the mutation-selection codon models, we focus on the structurally constrained (SC) models [4-7] which attempt to explicitly link a protein's tertiary structure to the evolution of its sequence. They consider that a protein is under a purifying selection maintaining a stable and constant tertiary structure. Importantly, and unlike most probabilistic models currently used in molecular evolutionary studies, SC models are explicitly site-interdependent, and therefore, require complex Monte Carlo methods to be implemented and applied to empirical data [3,4,8].

In SC models, the fixation probability of a given mutation depends on a score function assessing the adequacy of a sequence *s *to the tertiary structure of the protein, *c*. This score should be devised so that the fixation probability is low if the proposed mutation destabilizes the structure or complicates the folding process. Since Anfinsen's experiments [9], the relations between protein structure and sequence have been carefully studied and an intuitive approach consists in relying on first principles of protein thermodynamics, using all-atom force fields (e.g. AMBER [10], CHARMM [11]). However, in our case, the instantaneous rate of substitution (*R*_*ss'*_), and thus the structure/sequence score function, have to be computed for each possible nearest neighbor mutant, and for each substitution, along the entire evolutionary tree. Therefore, we need a fast computation of the fixation probability which precludes the use of all-atom force fields.

An attractive alternative is provided by knowledge-based (or statistical) potentials. They mimic the Boltzmann law [12-15] and usually rely on a coarse-grained description of the structure, implicitly integrating out the degrees of freedom of the side chains and thus avoiding the complexity and the computation requirements of all-atom force fields [16-23]. In addition, they are trained empirically from databases of natural proteins. This latter point is of particular interest in evolutionary studies, where we are interested in all aspects of the relations between sequence and structure prevailing in natural sequences, and not only in the specific problem of the thermodynamic stability. In this respect, one expects that learning potentials from native structure-sequence databases using blind machine learning methods will capture all such aspects.

Many statistical potentials have been proposed [12,14,15,19,24,25], either to predict the fold of a given sequence (*protein folding*) or to find a sequence or a set of sequences folding into a given tertiary structure (*protein design*). However, the same potential may not be best-suited to both goals since the spaces of optimization are very different: in the protein folding problem the search is done over the structure space, while in the protein design problem the search is done over the sequence space. The phylogenetic context described here is more akin to a protein design perspective, as the structure of the protein is assumed constant during evolution, representing a constraint under which the sequence is evolving.

Several methods have been developed to train statistical potentials in a protein design perspective [19,24,25]. In a previous work, we introduced a probabilistic framework for protein design purposes based on the maximum likelihood principle [26]. The likelihood we considered was the probability of the sequences *S *given their native structures *C *and the model parameters (here, the statistical potential parameters, *θ*), *P *(*S*|*C*, *θ*). This probability was then maximized with respect to the potential parameters (e.g. pairwise contact energy coefficients) by a gradient method. However, the probability *P *(*S*|*C*, *θ*) involves a normalizing factor, summing over all possible sequences, which cannot be analytically calculated. We thus had to resort to a Markov Chain Monte Carlo (*MCMC*) numerical procedure: at each step of the gradient descent, we generated a set of sequences by Gibbs sampling, conditional on the current values of the potential. This set of sequences was then used to estimate the gradient. The Gibbs sampling procedure was the limiting step of our algorithm, restricting the set of alternative potentials that we could explore and empirically test. The potentials we obtained using this method are called *joint *potentials hereafter.

Interestingly, Kuhlman and Baker [27] used a *leave-one-out *procedure to estimate a restricted set of parameters of a free physical energy function in order to do protein design. In this procedure, only one site of the protein is changed at a time, while the other positions are kept fixed in their native state. The procedure is thus similar to training a potential to recognize acceptable sequence variants, given the target structure, among all possible point mutants. The leave-one-out criterion seems to give good results. However, it has never been assessed against alternative methods. Here, we adapt the statistical framework we defined in [26] now using the leave-one-out definition of the likelihood to perform the gradient descent instead of the joint likelihood. We compare the potential parameters obtained by the two methods, and we establish that we can be highly confident in the results obtained using the leave-one-out likelihood. Overall, the leave-one-out procedure allows much faster computations while giving sensibly the same results as the joint one.

## Results

### Likelihood framework

As in [26], we formulate the problem in terms of a probabilistic model, considering a sequence *s *= (*s*_*i*_)_1..*n *_of length *n *according to a probability distribution *P *(*s*|*c*, *θ*), conditional on the conformation *c *and on a set of potential parameters *θ*. The parameters are estimated by maximizing the probability of observing a database of *N *independent sequence-structure pairs (, *C*), with , *C *= (*c*^*p*^)_*p *= 1..*N*_. Here,  is the *p*-th native sequence of the dataset, *n*_*p *_is the lenght of this sequence and *c*^*p *^is the native conformation associated with . In practice, a native sequence-structure pair corresponds to a protein taken from the PDB.

The probability that we want to maximize can be expressed as follows:

(2)

As a function of *θ*, this term can be seen as a likelihood. Hereafter, we define the methodology with one protein, but it can be easily generalized to a set of proteins.

Borrowing from [26], we set:

(3)

where *Y *is called the *normalization factor*, and *G*(*s*|*c*, *θ*) the *inverse potential*, defined as

(4)

where *E*(*s*|*c*, *θ*) is the statistical potential and *F *(*s*) is analogous to a free energy term and can be approximated using the *random energy model *[19,28-30]:

(5)

where *μ*_*a*_, *a *= {1..20} are unknown parameters, analogous to *chemical potentials *[26].

### Optimization method

#### Joint likelihood maximization

In our previous work [26], we defined a score function *ω *(|*c*, *θ*) as:

(6)

This score function should be minimized conditional to *θ*. Its gradient is:

(7)

where ⟨·⟩ stands for the expectation over sequences drawn from the probability defined by eq. 3. Given the size of the sequence space (20^*n*^), this expectation cannot be computed analytically, and therefore, in [26] we used a MCMC method to numerically estimate this expectation.

#### Leave-one-out likelihood maximization

We define for site *i, i *= 1..*n*, the leave-one-out probability

(8)

which is the probability of having an amino acid *a *at site *i*, in the context of the native sequence at all other sites (∀*j *≠ *i s*_*j *_= ). This leave-one-out probability can easily be obtained by a normalization over all possible twenty outcomes at site *i*:

(9)

We can write this probability for any amino acid *a*, and in particular for the native amino acid at site *i*,  i.e.. Taking the product over all positions *i *= 1..*n*, and by analogy with our previous definition of likelihood, we define the leave-one-out likelihood:

(10)

Note that this leave-one-out likelihood is normalized over the sequences, exactly as in the case of eq. 3. Therefore it yields a valid probability distribution over the sequence space. On the other hand, the probability depends not only on *c *and *θ*, but also, in some sense, on the native sequence itself. To make this point explicit, we make  appear on both sides of the conditioning bar.

We define the corresponding scoring function:

(11)

the gradient of which is immediately obtained (Additional File 1):

(12)

This gradient can be analytically calculated, at each step of a gradient descent. We note that the term corresponding to the normalization factor (the second term in eq. 12) can be seen as an expectation over the leave-one-out probability. It is thus analogous to the expectation appearing in the right hand of eq. 7. However, it is defined on a much more restricted universe (20·*n *states, compared to the 20^*n *^states in the case of the joint likelihood).

For implementing both methods, we used a simple form of potential [26], consisting in two terms: one related to contact interactions and the other to the solvent accessibility (see Methods).

### Potential optimization

We first run our leave-one-out method on *DS*_*l *_(see Methods). We consider that the optimization is complete when the overall maximum gradient is smaller than 10^-2^. This corresponds to a variation of 10^-6^, at most, in the value of the potential parameters. Using this stopping condition on the dataset *DS*_*l *_with empirically tuned general steps (e.g for the contact parameters:  = 10^-5 ^and for the solvent accessibility parameters:  = 10^-4^), we compare three different gradient descent methods (described in Methods): the simple gradient descent, the inertial gradient descent, and the controlled inertial gradient descent. The values of the parameters stabilized after 14,500 gradient steps for the simplest gradient descent, versus 1,500 gradient steps for the inertial gradient, and 1,200 gradient steps for the controlled inertial gradient. Concerning the last method, if we choose a different general step (e.g.  = 10^-3 ^and  = 10^-2^) the procedure automatically reaches the optimal step for that dataset. At the beginning of the optimization procedure, the inertial component of the gradient greatly speeds up the optimization, but is automatically deactivated when the values of the potential parameters are near the optimum, thus avoiding the numerical instabilities usually observed using less adaptive gradient methods.

**Figure 1:**
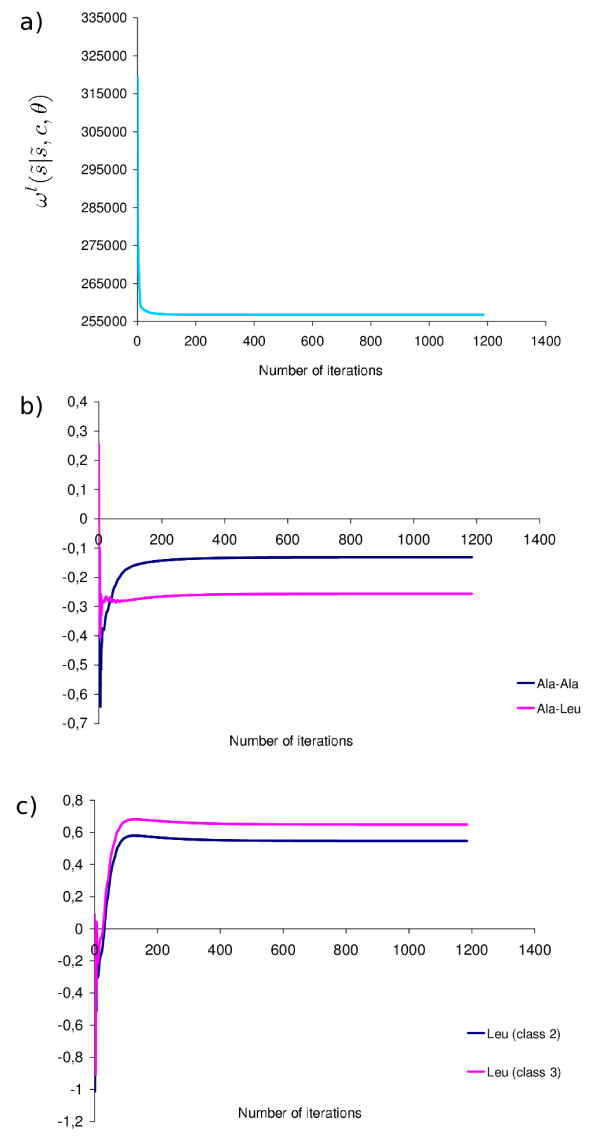
**Convergence of the optimization procedure**. Evolution of (a) the score function, (b) contact potential parameters and (c) accessibility potential parameters, for the dataset *DS*_*l*_, using the controlled inertial gradient descent.

**Figure 2:**
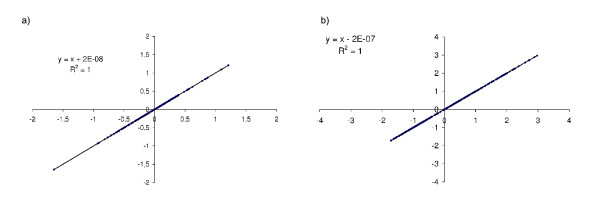
**XY comparisons of the leave-one-out potential parameters**. XY comparisons of two independent runs on the same dataset *DS*_*l *_for (a) contact and (b) solvent accessibility potential parameters respectivly.

Independent runs from different and randomly chosen initial values for the parameters of the leave-one-out potential (*θ*^*l*^), lead to the same final values of *ω*^*l*^(|, *c*, *θ*) (fig. 1) and of the potential parameters (fig. 2). These computations were done with the three gradient descent methods, and resulting always in the same final values, which suggests that, in the present case, we do not have local minima in the space of parameters. Similarly, the potential parameters obtained by two independent runs on the same dataset are very similar, indicating that our stopping condition is sufficient to have a good precision in our estimates (Additional file 2). In fig. 1 we have also represented the evolution of some parameters of the potential during optimization. We can see that the values of these parameters oscillate at the beginning of the gradient descent and then reach their optimal values. This behavior is caused by the evolution of the other parameters, as they influence each other during optimization. The complete series of parameter values obtained by our optimization method are presented in the additional file 3.

**Figure 3:**
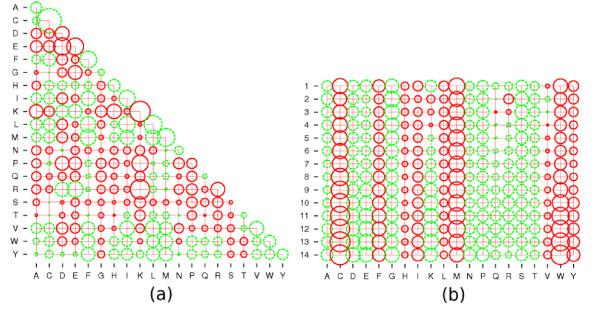
**Validation of the potential parameters**. Bubble plot representations of (a) contact potential parameters and (b) accessibility potential parameters obtained upon the dataset *DS*_*l*_. Negative values are plotted in green while positive values are plotted in red.

The contact potentials obtained with the leave-one-out optimization criterion make sense from a biological point of view (fig. 3): as expected, favorable interactions between amino acids in the contact potentials are represented by large negative value (e.g. the Cysteine-Cysteine contact energy, fig. 3), and by large positive value for unfavorable interactions (e.g. the Lysine-Lysine or Lysine-Arginine interactions, which are electrostatically repulsive). Concerning the accessibility potentials, it is important to note that we are working in a protein design context (i.e. we are evaluating the fitness of alternatives amino acids in a given accessibility class). Accordingly, the accessibility potentials have to be interpreted row-wise. If one wants to compare the accessibility potentials between classes for a given amino acid (i.e. in a protein folding perspective), one solution is to remove the logarithm of the frequency of the accessibility classes to each potential (additional file 4). Also, note that there is a lack of identifiability between *α *and *μ*, which has been be resolved by including the chemical potentials in the accessibility terms.

### Complexity

In our previous work, we had to use a MCMC protocol to numerically evaluate the derivative of the gradient (see. eq. 7), which was a computationally demanding task. At each step of the gradient descent, we had to sample a set of sequences by Gibbs sampling, under the current values of the parameters, so as to numerically estimate the gradient of the log-likelihood.

To compare the joint and the leave-one-out potentials, we first define an elementary calculation as the evaluation of the *inverse *potential at a particular site *i *for one particular amino acid *a *(what we called *G*_*i*_(*s*_*i *_= *a*|, *c*, *θ*), eq. 9). This calculation has to be made in both cases. It is explicitly defined in the leave-one-out procedure (eq. 10), and is implicitly used in the joint context: an elementary step of the Gibbs sampling algorithm consist in computing, at a given site *i *the leave-one-out probability (eq. 9) for each possible amino-acid at this site, conditional on the rest of the sequence, and to choose the new aminoacid at site *i *according to these probabilities. Performing such an elementary update for every site in turn corresponds to one Gibbs sampling sweep and represents 20·*n *elementary computations. A reliable estimate of the joint expectation requires *K *sweeps (burn in included) and so, for a gradient step, we need *K*·*n*·20 elementary calculations (in practice, *K *≃ 1,000).

In the case of the leave-one-out potential, we only have to make the equivalent of one sweep to exactly compute the gradient (eq. 12). Thus, we only need *n*·20 elementary calculations for a gradient step, which thus represents a 1,000-fold increase in computational speed compared to the joint method. In practice, and after the addition of the acceleration of the gradient descent, it took about one week to have a good estimate when we used the joint method, versus less than fifteen minutes when using the leave-one-out approach.

### Potentials are indistinguishable

**Figure 4:**
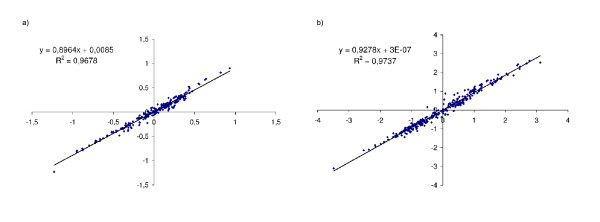
**XY comparisons of the leave-one-out and joint potential parameters**. XY comparisons between the two potentials (optimized on the same dataset *DS*_*j*_), with, in X-axis the leave-one-out potential, and in Y-axis the joint potential. (a) represents the correlation between the contact potential parameters, and (b) the correlation between the accessibility potential parameters.

We applied the two optimization procedures (joint and leave-one-out) to the same dataset *DS*_*j*_, and found a high correlation between the two resulting potentials (fig. 4). The correlation coefficient *R*^2 ^was about 0.96779 for the contact potential parameters and about 0.97374 for the accessibility potential parameters. For comparison, we applied the leave-one-out procedure on the two datasets DS1 and DS2 (see additional file 2) and found a correlation coefficient of 0.9477 for the contact parameters and of 0.9596 for the accessibility parameters, indicating that the difference between the joint and the leave-one-out potentials is small compared to the sampling error due to the finite size of the training set. Altogether, the leave-one-out method appears to be a fast and reliable optimization procedure, yielding potentials that are virtually indistinguishable from those obtained under the joint method. As presented in [26], the contact potentials present a correlation (*R*^2 ^= 0.6565) with those of Miyazawa and Jernigan [13].

### Phylogenetic evaluation

In eq. 1, we defined the substitution process of the SC model as a process depending on a mutation rate and a fixation probability. There are many ways the fixation probability could be expressed. Here, we do as in Robinson et al [4] and assume that this probability depends only on the potential difference (Δ*G*) between the original and the mutated sequences. Let us denote by *s*_*nuc *_and , two sequences which differ only by a nucleotide, and *s*_*aa *_and , the corresponding amino acid sequences (which may be identical due to codon synonymy). Then, the rate of substitution between *s *and *s' *is:

(13)

where  is the mutation term depending only on the two sequences *s*_*nuc *_and .  is the energy difference between *s*_*aa *_and , and *β *≥ 0 can be considered as the strength of the structure-sequence constraint enforced by the model. Thus, a negative (resp. positive) Δ*G *means that the mutation is more (resp. less) likely to be accepted than a purely neutral (e.g. synonymous) mutation.

Note that the substitution process defined by eq. 13 is reversible and has a stationary distribution defined by:

(14)

where ∏_0_(*s*_*nuc*_) is the stationary distribution induced by the pure mutation process (). Given the way our potentials are optimized (see eq. 3 and 9) and assuming that natural sequences are sampled at equilibrium from the process defined by eq. 13, we then expect that the optimal value of *β *should be close to 0.5. In the following, we explore the entire range *β *∈ [0, 1].

We denote by  the SC model defined using the leave-one-out potential and  the SC model defined using the joint potential; the two models depend on *β*. Obviously, when *β *= 0,  =  = *SC*_0_, and the model reduces to a pure mutation model which will be considered as our reference model.

**Figure 5:**
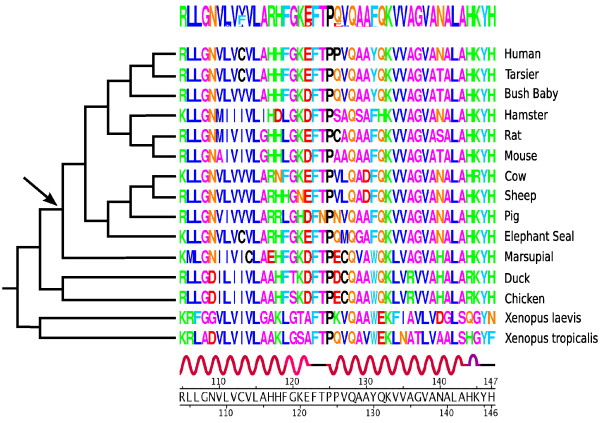
**Logo profile of the mammalian ancestral globin sequence**. The node is marked by an arrow. The translated sequences of the true alignment are displayed along with the secondary structure of the structure PDB code 4HHBB.

We implemented our potential in the SC model as described in [3] and applied it to the GLOBIN15-144 dataset, with an underlying mutational specification inspired by the codon model in [31] and denoted as MG in [3]. This MCMC framework allows one to obtain a sample of parameter values and substitutional histories along the tree, drawn from the posterior distribution under the  model. Such a sample can then be marginalized over quantities of interest. Here, we briefly illustrate the approach by displaying the logo of the reconstructed mammalian ancestor hemoglobin sequence (fig. 5).

**Figure 6:**
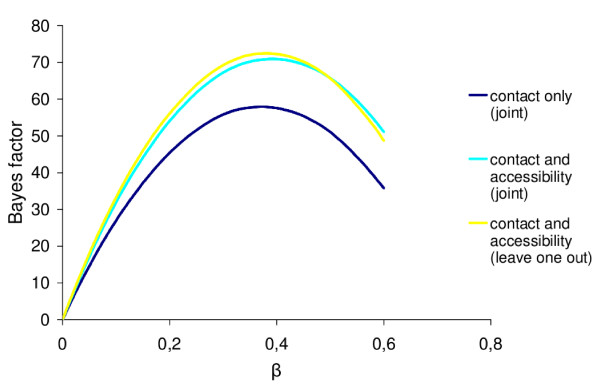
**Bayes factor**. Curves representing the Bayes factor as a function of *β*, with  (in yellow),  (in light blue) and  (in dark blue), for the dataset BGLOBIN15-144.

Since the leave-one-out procedure can be seen as an approximate but faster training method, compared to the joint method developed previously, we evaluated its impact on model fit via Bayes factors evaluations (see Methods). In this section we consider the three versions of the SC model, , based on a contact + accessibility leave-one-out potential, , based on a contact + accessibility joint potential, and  based on a contact only joint potential. As explained in the methods, in the present case, the thermodynamic integration method yields a complete fitness curve (fig. 6) of each model (i.e. a curve representing the Bayes factor of each model against the reference model, as a function of *β*). In this way, we can readily spot the optimal value of *β *under each model, and report the Bayes factors under this optimal value (table 1).

**Table 1:** The natural logarithm of the Bayes factors.

	**ADH23-254**	**CALM36-444**	**GLOBIN15-144**	**LYS25-134**
	[74.748-75.032]	[149.819-149.929]	[57.953-58.135]	[11.5-11.968]

	[102.666-102.766]	[161.340-161.491]	[70.666-70.948]	[26.287-26.417]

	[102.977-103.115]	[158.679-158.858]	[72.485-72.872]	[29.545-29.852]

optimal *β*	[0.387-0.397]	[0.371-0.383]	[0.450-0.498]	[0.179-0.249]

As can be seen from fig. 6 and table 1, the models based on the joint and the leave-one-out potentials have a very similar fit across the whole range of value of *β *that we tested. Interestingly, in all but one cases, the Bayes factor appears to be slightly in favor of the leave-one-out potential, although the differences are not significant. As a point of comparison, we also measured the fit of the contact only potential (joint method), to illustrate that the difference between the joint and the leave-one-out methods is small compared to the differences observed between the alternative forms of statistical potential that we would like to empirically compare (see [26] for an evaluation of the relative contribution of each potential component to the fitness of the model).

## Discussion

In a previous work [26], we defined a statistical framework for protein design, using the maximum likelihood principle, with the aim of devising statistical potentials to be used in phylogenetic studies. However, the optimization procedure we introduced at that time requires a MCMC protocol to cope with the proportionality constant entailed by the normalization of the probability over the sequence space. Here, we introduce a different likelihood, which we called leave-one-out, to optimize the potentials. A similar procedure was previously used by Kuhlman and Baker [27], but was not statistically assessed against alternative procedures. We found in this work that the joint and the leave-one-out potentials are virtually indistinguishable, both by direct comparison and by Bayes factor evaluation in a phylogenetic context.

We note that the optimal *β *for the  model is not 0.5, as one may expect given the way our potentials were normalized (see eq. 3, 6 and 13). Several explanations can be proposed. First, strictly speaking, this expectation is valid under the joint procedure, and not under the leave-one-out procedure. But the very high similarity between the two resulting potentials, and the fact that a similar phenomenon (*β *≠ 0.5) can be observed also under a potential optimized using the joint method [3] do not favor this explanation. Alternatively, it may appear at first that this could be due to the fact that the underlying mutation model (the *Q*^*mut *^matrix in eq. 13) was not explicitly taken into account when optimizing the potential (so that the chemical potentials implicitely include a mutational component), whereas our phylogenetic model does involve an explicit mutational process. In this sense, in the phylogenetic analysis, there is a potentially (partially) redundant modeling of mutational features, in having explicit parameters devoted to these, in combination with the use of the SC setting. This might explain the optimal value of *β *lower than 0.5. The phenomenon may also be the result of model violations, which are very likely to be present given the simple form of the potentials. Finally, it is also likely that the mutation pressure, or the selection strength (represented by *β*) is not the same for each protein. Accordingly, two possible improvements to the method can thus be proposed here: the first is to optimize the potential while allowing for different values of *β *for each protein or each family of protein. The second is to cluster proteins into classes, and optimize a potential specifically for each class.

## Conclusion

Apart from these two possible improvements, many other directions of research should now be explored: alternative functional forms for the potential should be implemented and empirically tested. Several methods accounting for negative design, through the use of explicit decoys [18] such as the use of a normalized energy gap between a native structure and misfolded structures [32], or using variational methods [19], also deserve further investigation. The supervised learning described here depends on structure-sequence pairs. In the present case, we have used native pairs, but this could be relaxed by taking a set of structures (e.g. obtained by molecular dynamics) as the reference structure or by taking a set of homologous sequences instead of a unique sequence [33]. A more appealing method would consist in doing the optimization directly within the phylogenetic context. Importantly, the fact that the leave-one-out procedure is much faster than the joint method (in the present case, roughly by a factor 1,000), has obvious practical consequences, as it allows a much larger diversity of alternative models and methods to be tested.

## Methods

### Gradient descent

When performing a gradient descent, several methods can be used. We expose here the three gradient descent methods that we compared. In all cases, the method rely on a cyclical updating of parameter values, where, given the values of parameters at the *m*^th ^cycle, which we write as *θ*^(*m*)^, the update is given by:

(15)

The increment, Δ*θ*^(*m*+1)^, is conditional to the scoring function, that we simply denote in this part as *ω *(*θ*^(*m*)^).

#### Fixed step gradient

This is the simplest form of the gradient descent. We write:

(16)

where *δ*_*grad *_is the fixed step of the gradient descent. Even though this formalism is simple, the choice of the step is not trivial. Indeed, if the step is too large, the values of the potential will oscillate around the optimal values. Conversely, if the step is too small, the gradient descent will be too slow.

#### Inertial gradient

To reduce the optimization time, another method of gradient descent was developed, based on an analogy with the physical phenomenon of inertia.

(17)

*δ*_*iner *_is the damping rate of the inertial component, 0 ≤ *δ*_*iner *_< 1. If *δ*_*iner *_= 0, eq. 17 reduces to the case of the simple gradient. In practice, we set *δ*_*iner *_equal to 0.9.

However, there is a drawback when taking into account the previous variation of the parameters: when the directions of the gradient change, the inertial part of the gradient brings the parameters too far beyond the maximum. In addition, the gradient step *δ*_*grad *_has to be small enough so that the values of the potential do not oscillate around the optimal values, as in the case of the fixed step gradient.

#### Controlled inertial gradient

To avoid these two drawbacks, we define here a controlled inertial gradient descent formalism. Specifically, let us define:

(18)

(19)

The decision procedure can thus be described as follows (see additional file 5). First, we test if the addition of Δθ* (derivative component and inertial component) to the actual values of parameters *θ*^(*m*) ^gives a higher likelihood than *θ*^(*m*)^. If it does, then the step corresponds to a classical step of the inertial gradient descent. Otherwise, the algorithm tests if the addition to *θ*^(*m*) ^of the derivative component (Δ*θ*^•^) only gives a higher likelihood than the actual values. If it does, the step corresponds to a classical gradient descent. Otherwise, we retry a simple gradient descent with a smaller *δ*_*grad*_.

The above procedure has two advantages. The first is the speed-up offered by the inertial component, when its addition has a positive influence on the likelihood. The second advantage is that the last part of the algorithm automates the search for an optimal value of the steps, and avoids both oscillations of *θ *around the optimum, and a slow gradient descent.

### Statistical potentials

We used the same statistical potential function as in our previous work [26]. The (pseudo) energy score consists of two terms:

(20)

The first term represents the contact free energy (defined between sidechain centers): Δ_*ij *_= 1 if *i *and *j *are closer than the cutoff distance (here 6.5 Å), and *ε*_*ab *_represents the contact potential between amino acids *a *and *b*. The second term represents the accessibility free energy: *ν*_*i *_is the accessibility class of the site *i *and  is the solvent accessibility potential of the amino acid *a *when placed into the accessibility class *d *(*d *= {1..*D*}), where *D *is the number of accessibility classes.

We use the *random energy model *principle to approximate *F *(*s*) (eq. 5), so that the inverse potential becomes:

(21)

As in our previous work we fix the constraints:

(22)

(23)

(24)

since *G*(*s*|*c*, *θ*) is invariant under the following transformations ,  and . However, there is an additional lack of identifiability between *a *and *μ*, which can be resolved by including the chemical potentials in the accessibility terms. Indeed, the *μ*_*a *_terms can be seen as an additive constant to each accessibility term for a given accessibility class (see additional file 6). In the present case, our final inverse potential is therefore:

(25)

and our set of parameters for the statistical potential will thus consist of:

(26)

### Bayes factor evaluation

In a Bayesian statistical framework the method of choice for comparing models is to compute Bayes factors. The Bayes factor between two models is defined as the ratio of their respective marginal likelihood. The case *B*(*SC*_0_, ) > 1 (resp. *B*(*SC*_0_, ) < 1) is considered as an evidence in favor of (resp. against) the  model. We write the Bayes factor between *SC*_0 _and  as:

(27)

where *A *corresponds to the data, composed by an alignment of coding nucleotide sequences and a topology and

(28)

Here we compute Bayes factors by thermodynamic integration (or *path sampling*) as described in [3]. The procedure consists in sampling along a continuous path between *SC*_0 _and  through a set of slight changes in the value of *β*. In fact, this procedure provides a complete curve representing the fit of the model as a function of *β*. Sampling from *β *= 0 to *β *= *β*_*max *_and from *β *= *β*_*max *_to *β *= 0 gives two different curves for the logarithm Bayes factor, which we used as an internal check of the reliability of the method (not shown).

### Datasets

#### Optimization datasets

The datasets are made of proteins (structure-sequence pairs) culled from the PDB, with less than 25% of mutual sequence identity and a resolution better than 2 Å [34]. This sequence homology percentage and the size of the database avoid possible bias that could be induced by related proteins. To compare the joint and leave-one-out potentials, we used the dataset on which we previously estimated the joint potentials, *DS*_*j*_. This dataset is made of 441 proteins and 98,155 sites [26]. We also consider a dataset *DS*_*l *_(made of 3,363 proteins and 835,717 sites) which was split into two subsets: *DS*1 (1,691 proteins and 419,208 sites), and *DS*2 (1,672 proteins and 416,509 sites). To determine the accessibility classes, we first compute the solvent accessibility area using Naccess 2.1 [35] and partitioned the resulting values into classes [26].

#### Phylogenetic Datasets

The SC model was applied to 4 distinct multiple sequence alignments: GLOBIN15-144, LYSIN25-134, ADH23-254 and CALM33-444. GLOBIN15-144 is made of 15 vertebrates sequences of the *β*-globin gene (taken from the original dataset from [36]), with a protein structure defined by the PDB file 4HHB and a tree topology estimated using Phylobayes 3.1c [37] (which is consistent with the tree topology described in [38]). LYSIN25-134 is made of 25 Abalone sperm lysin sequences [39], with a protein structure defined by the PDB file 1LYS and the tree topology previously defined by [39]. ADH23-254 is made of 23 alcohol dehydrogenase sequences taken form Drosophila [36], with a protein structure defined by the PDB file 1A4U and the tree topology previously defined by [36]. CALM36-444 is made of 36 calmodulin sequences taken from eukaryotes, with a protein structure defined by the PDB file 1CFD and the tree topology estimated using phyML [40] under the model JTT + F + Γ [41,42].

## Authors' contributions

CB implemented the leave-one-out and gradient descent methods described here and performed the run of all the experiments. CLK implemented the data pre-processing methods. NR implemented the phylogenetic framework. NL set up the theoretical framework and directed the overall project. All the authors co-wrote the manuscript and approved the final manuscript.

## Supplementary Material

Additional file 1 Derivatives of the potential parameters. Click here for file

Additional file 2 XY-comparison of the leave-one-out potentials estimated from two independent datasets: (a) and (b) two independent runs on *DS*1 (X-axis) and *DS*2 (Y-axis) for contact and accessibility potentials respectivly. Click here for file

Additional file 3 Contact potentials and solvent accessibility potentials written in an alphabetical order. Click here for file

Additional file 4 Bubble plot of the solvant accessibility potential where we remove from each potential the corresponding natural logarithm frequency of the accessibility class. Click here for file

Additional file 5 Controlled inertial gradient algorithm. Click here for file

Additional file 6 Inclusion of *μ*_*a *_in the accessibility terms. Click here for file
