# Supplementary material for: Fast optimization of statistical potentials for structurally constrained phylogenetic models
Source: BMC Evol Biol. 2009 Sep 9;9:227. doi: 10.1186/1471-2148-9-227 (PMC2754480; doi:10.1186/1471-2148-9-227)
Supplement: Additional file 1 — Derivatives of the potential parameters. [file 1471-2148-9-227-S1.pdf]

We found the derivative of the gradient:

$$\frac{\partial \omega(\tilde{s}|\tilde{s}, c, \theta)}{\partial \theta} = \sum_{i=1..n} \left(-\frac{\partial F(\tilde{s}_i|\tilde{s}_{\setminus i}, c, \theta)}{\partial \theta} + \sum_{a=1..20} p_i(a) \frac{\partial F(a|\tilde{s}_{\setminus i}, c, \theta)}{\partial \theta} \right). \quad (1)$$

As for the joint criterion, the derivatives can be immediately calculated and become:

$$\frac{\partial \omega(\tilde{s}|\tilde{s}, c)}{\partial \varepsilon_{ab}} = -n_{ab} + \sum_{1 \le i < j \le n} \Delta_{ij} \left(P_i(a) \cdot D(b, s_j) + P_i(b) \cdot D(a, s_j) \right), \quad (2)$$

$$\frac{\partial \omega(\tilde{s}|\tilde{s}, c, \theta)}{\partial \alpha_a^d} = -l_a^d + \sum_{1 \le i \le n} P_i(a) D'(d, \nu_i), \quad (3)$$

where n_{ab} is the number of contacts between amino acids a and b observed in the database and l_a^d is the number of amino acids of type a belonging to the accessibility class d. $D(k, l) = 1$ if k and l are the same amino acid, and $D'(d, e) = 1$ if d and e are the same accessibility class. We can see these equations as

$$\frac{\partial \omega(\tilde{s}|\tilde{s}, c, \theta)}{\partial \varepsilon_{ab}} = -n_{ab} + \langle n_{ab} \rangle_l, \quad (4)$$

$$\frac{\partial \omega(\tilde{s}|\tilde{s}, c, \theta)}{\partial \alpha_a^d} = -l_a^d + \langle l_a^d \rangle_l, \quad (5)$$

which are the same formulation that were used for the derivatives for the joint criterion.
