# Supplementary material for: Fast optimization of statistical potentials for structurally constrained phylogenetic models
Source: BMC Evol Biol. 2009 Sep 9;9:227. doi: 10.1186/1471-2148-9-227 (PMC2754480; doi:10.1186/1471-2148-9-227)
Supplement: Additional file 5 — Controlled inertial gradient algorithm. [file 1471-2148-9-227-S5.pdf]

Algorithm 1: Choice algorithm for the gradient descent

Data: A set of values of parameters $\theta^{(m)}$, a gradient $d\Omega = \frac{\partial \omega(\tilde{s}|\tilde{s},c,\theta)}{\partial \theta}$, a step δ_{grad}, a value $0 < r < 1$.

Results: A set of values of parameters $\theta^{(m+1)}$ like $\omega^l(\tilde{s}|\tilde{s},c,\theta^{(m+1)}) < \omega^l(\tilde{s}|\tilde{s},c,\theta^{(m)})$.

begin
- $\theta^{(m+1)} := \theta^{(m)}$
- **while** $\theta^{(m+1)} = \theta^{(m)}$ **do**
  - $\theta^\star = \theta^{(m)} - \delta_{grad} d\Omega - \Delta\theta^{(m)}$
  - **if** $\omega^l(\tilde{s}|\tilde{s},c,\theta^\star) < \omega^l(\tilde{s}|\tilde{s},c,\theta^{(m)})$ **then**
    - $\theta^{(m+1)} = \theta^\star$
  - **else**
    - $\theta^\star = \theta^{(m)} + \delta_{grad} d\Omega$
    - **if** $\omega^l(\tilde{s}|\tilde{s},c,\theta^\star) < \omega^l(\tilde{s}|\tilde{s},c,\theta^{(m)})$ **then**
      - $\theta^{(m+1)} = \theta^\star$
    - **else**
      - $\delta_{grad} = \delta_{grad} * r$

end
