# Supplementary material for: Fast optimization of statistical potentials for structurally constrained phylogenetic models
Source: BMC Evol Biol. 2009 Sep 9;9:227. doi: 10.1186/1471-2148-9-227 (PMC2754480; doi:10.1186/1471-2148-9-227)
Supplement: Additional file 6 — Inclusion of μa in the accessibility terms. [file 1471-2148-9-227-S6.pdf]

Keeping the constraints defined by

$$\sum_{1 \le a \le 20} \mu_a = 0, \tag{1}$$

$$\sum_{1 \le a \le 20} \sum_{1 \le b \le 20} \varepsilon_{ab} = 0, \tag{2}$$

$$\sum_{1 \le a \le 20} \alpha_a^d = 0, d = \{1..D\}, \tag{3}$$

if we define

$$\forall d \quad \alpha'^d_{a_k} = \alpha^d_{a_k} + J \quad \text{and} \quad \mu'_{a_k} = \mu_{a_k} - J, \tag{4}$$

and

$$\forall d \quad \alpha'^d_{a_l} = \alpha^d_{a_l} - J \quad \text{and} \quad \mu'_{a_l} = \mu_{a_l} + J, \tag{5}$$

then

$$\begin{aligned} G'(s,c) &= \sum_{1 \le i < j \le n} \varepsilon_{s_i s_j} + \sum_{1 \le i \le n} \alpha'^{\nu_i}_{s_i} + \sum_{1 \le i \le n} \mu'_{s_i} && (6) \\ &= \sum_{1 \le i < j \le n} \varepsilon_{s_i s_j} + \sum_{1 \le i \le n} \alpha^{\nu_i}_{s_i} - J + J + \sum_{1 \le i \le n} \mu_{s_i} + J - J && (7) \\ &= G(s,c). && (8) \end{aligned}$$

So, repeting this iteratively from $(k = a_1, l = a_2)$ to $(k = a_{19}, l = a_{20})$, then

$$\forall a, 1 \le a \le 20, \quad \mu_a = 0. \tag{9}$$

And thus, $\mu_a, a = \{1..20\}$ terms can be all included in the accessibility terms.
